# Impact of Obstructive Sleep Apnea and Sympathetic Nervous System on Cardiac Health: A Comprehensive Review

**DOI:** 10.3390/jcdd11070204

**Published:** 2024-06-30

**Authors:** Antonino Maniaci, Salvatore Lavalle, Federica Maria Parisi, Marco Barbanti, Salvatore Cocuzza, Giannicola Iannella, Giuseppe Magliulo, Annalisa Pace, Mario Lentini, Edoardo Masiello, Luigi La Via

**Affiliations:** 1 Department of Medicine and Surgery, University of Enna “Kore”, 94100 Enna, Italy; antonino.maniaci@unikore.it (A.M.); marco.barbanti@unikore.it (M.B.); 2 Department of Medical, Surgical Sciences and Advanced Technologies “GF Ingrassia” ENT Section, University of Catania, 95123 Catania, Italy; federicamariaparisi@gmail.com (F.M.P.); s.cocuzza@unict.it (S.C.); 3 Otorhinolaryngology Department, Sapienza University of Rome, Policlinico Umberto I, Viale del Policlinico, 00161 Rome, Italy; giannicola.iannella@uniroma1.it (G.I.); giuseppe.magliulo@uniroma1.it (G.M.); annalisa.pace@uniroma1.it (A.P.); 4 ASP Ragusa-Hospital Giovanni Paolo II, 97100 Ragusa, Italy; marlentini@tiscali.it; 5 Radiology Unit, IRCCS San Raffaele Scientific Institute, Via Olgettina 60, 20132 Milan, Italy; 6 Department of Anesthesia and Intensive Care, Azienda Ospedaliero Universitaria Policlinico “G. Rodolico–San Marco”, 95123 Catania, Italy

**Keywords:** sleep apnea, sympathetic nervous system, cardiac risk, cardiovascular health, sympathetic activation

## Abstract

A prevalent condition linked to an elevated risk of cardiovascular disease is sleep apnea. This review examines the connections between cardiac risk, the sympathetic nervous system, and sleep apnea. The increased risk of hypertension, arrhythmias, myocardial infarction, and heart failure was highlighted in the pathophysiology of sleep apnea and its effect on sympathetic activation. It is also important to consider potential processes such as oxidative stress, inflammation, endothelial dysfunction, and autonomic imbalance that may relate sleep apnea-induced sympathetic activation to cardiac risk. With implications for creating innovative diagnostic and treatment approaches to lessen the cardiovascular effects of sleep apnea, the goal of this investigation is to improve the understanding of the intricate link between sympathetic activity, cardiac risk, and sleep apnea. This study aimed to clarify the complex relationship between cardiovascular health and sleep apnea by synthesizing the available research and highlighting the crucial role played by the sympathetic nervous system in moderating this relationship. Our thorough investigation may have important therapeutic ramifications that will direct the creation of focused therapies to enhance cardiovascular outcomes in sleep apnea sufferers.

## 1. Introduction

Obstructive sleep apnea (OSA) is a prevalent sleep disorder characterized by recurrent episodes of the partial or complete obstruction of the upper airway during sleep, leading to intermittent hypoxia and sleep fragmentation [1]. The prevalence of OSA in the general adult population is estimated to be 9–38%, with higher rates observed in older individuals, males, and those with obesity [2,3]. OSA has a significant impact on overall health and quality of life, as it is associated with excessive daytime sleepiness, cognitive impairment, and an increased risk of accidents [4,5]. Moreover, the economic burden of OSA is substantial, with annual costs attributed to OSA and its comorbidities estimated to be in the billions of dollars in the United States alone [6]. OSA has been identified as a significant risk factor for various cardiovascular complications [7]. Patients with OSA have a higher prevalence of hypertension compared to the general population, and the severity of OSA is positively correlated with the degree of blood pressure elevation [8,9]. Additionally, individuals with OSA are at an increased risk of developing arrhythmias, particularly atrial fibrillation, which may be attributed to recurrent episodes of hypoxia and sympathetic activation [10,11]. Furthermore, OSA is associated with a higher incidence of myocardial infarction and heart failure, suggesting that it may contribute to the development and progression of these life-threatening conditions [12,13]. Several potential mechanisms have been proposed to explain the link between OSA and cardiovascular complications, including intermittent hypoxia, oxidative stress, inflammation, and endothelial dysfunction [14,15]. The sympathetic nervous system plays a crucial role in maintaining cardiovascular homeostasis by regulating heart rate, blood pressure, and vascular tone [16]. Sympathetic activation occurs in response to various stimuli, such as physical activity, stress, and changes in blood pressure or oxygenation [17]. When activated, the sympathetic nervous system increases heart rate, constricts blood vessels, and raises blood pressure to ensure the adequate perfusion of vital organs [18]. However, chronic or excessive sympathetic activation has been implicated in the pathogenesis of several cardiovascular disorders, including hypertension, heart failure, and arrhythmias [19,20]. Elucidating the interplay between OSA and sympathetic nervous system activation is essential for understanding the mechanisms underlying the increased cardiac risk in individuals with OSA and for developing targeted therapeutic interventions to mitigate these risks [21]. This comprehensive review aims to provide a detailed analysis of the complex relationship between obstructive sleep apnea, sympathetic nervous system activation, and cardiac risk. By synthesizing the current literature on this topic, we intend to enhance our understanding of the pathophysiological mechanisms linking OSA to cardiovascular complications and to identify potential avenues for future research and therapeutic interventions.

## 2. Materials and Methods 

In order to comprehensively discuss the effects of obstructive sleep apnea (OSA) and the sympathetic nervous system (SNS) on cardiac health, we conducted a meticulous and methodical study of the existing literature. We conducted a comprehensive search across multiple databases, such as PubMed, Web of Science, Scopus, and Google Scholar, to include all the pertinent papers until May 2024. The search phrases we used included keywords and Medical Subject Headings (MeSH) that were relevant to OSA, SNS, and other aspects of cardiac health, such as cardiovascular illness, heart disease, autonomic nervous system, hypertension, and cardiac arrhythmias. This research included the collection of quantitative data on cardiac outcomes, such as heart rate variability, blood pressure, and the occurrence of cardiovascular events. In contrast, we specifically did not include publications in languages other than English, research using animals, and sources that have not undergone peer review, such as case reports, editorials, and comments. Additionally, we omitted the studies that did not clearly investigate the relationship between OSA and SNS, and cardiac health. The process of extracting and managing data was carried out with great attention to detail. Two autonomous reviewers evaluated the titles and abstracts of all the identified papers. Individuals who were considered potentially eligible conducted a comprehensive evaluation of the entire text. We employed a uniform template for extracting data, encompassing information on research attributes, the demographic characteristics of the population, the definitions and measurements of OSA and SNS activity, cardiovascular health outcomes, as well as the primary discoveries and conclusions. To synthesize the data, we performed a narrative synthesis to highlight the findings on the effects of OSA and SNS activity on cardiac health.

## 3. What Is the Pathophysiology of Obstructive Sleep Apnea and Sympathetic Nervous System Activation in OSA?

**Figure 1:**
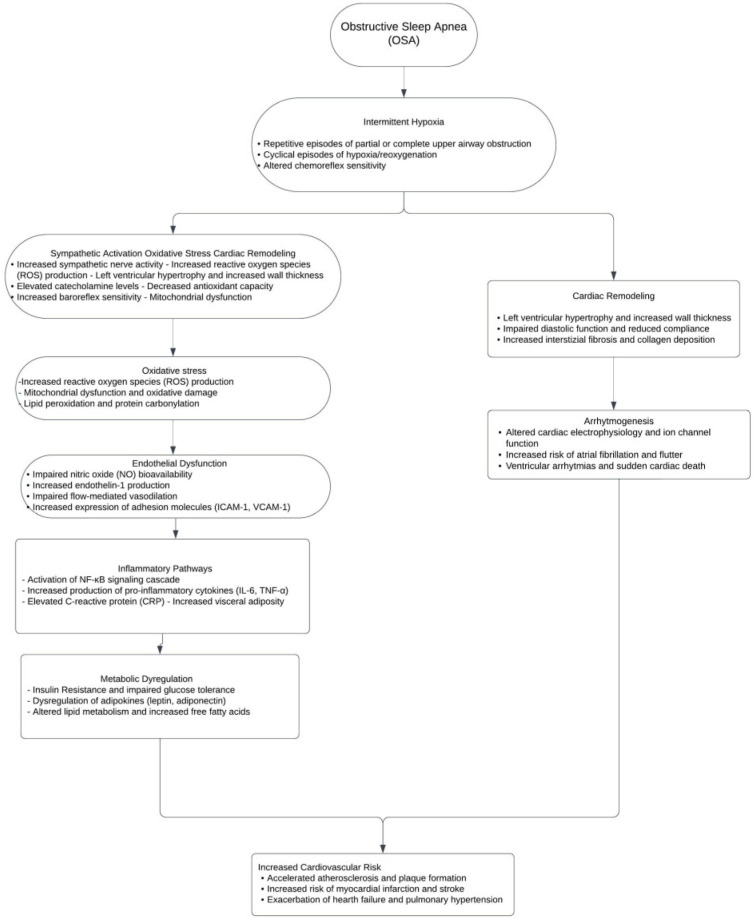
Flowchart of pathophysiological mechanisms determining increased cardiovascular risk in patients with OSA. Abbreviations: OSA, obstructive sleep apnea; ROS, reactive oxygen species; NO, nitric oxide; NF-κB, Nuclear Factor kappa B; IL-6, Interleukin-6; TNF-α, Tumor Necrosis Factor alpha; CRP, C-Reactive Protein; VCAM-1, Vascular Cell Adhesion Molecule 1.

Obstructive sleep apnea is characterized by repetitive episodes of the partial or complete collapse of the upper airway during sleep, leading to reduced or absent airflow despite ongoing respiratory efforts [22]. The pathophysiology of OSA involves a complex interplay between anatomical factors, such as craniofacial structure and soft tissue volume, and neuromuscular factors, including the responsiveness of upper airway dilator muscles (Figure 1) [23]. During sleep, the reduction in muscle tone and the loss of wakefulness drive can result in the narrowing or closure of the pharyngeal airway, particularly in individuals with predisposing anatomical features [24]. The recurrent episodes of airway obstruction in OSA lead to intermittent hypoxia and reoxygenation, a pattern of repeated oxygen desaturation followed by rapid reoxygenation when breathing resumes [25]. This cyclical pattern of hypoxia and reoxygenation has been shown to activate various cellular and molecular pathways, including oxidative stress, inflammation, and sympathetic activation [26]. Intermittent hypoxia has been implicated in the development of endothelial dysfunction, systemic inflammation, and metabolic dysregulation, all of which may contribute to the increased cardiovascular risk associated with OSA [27,28]. Obstructive sleep apnea is also associated with recurrent arousals from sleep, which occur in response to increased respiratory effort and the need to restore airway patency [29]. These arousals lead to sleep fragmentation and reduced sleep quality, contributing to the excessive daytime sleepiness and cognitive impairment commonly observed in patients with OSA [30]. Moreover, sleep fragmentation has been shown to activate the sympathetic nervous system and alter the balance between sympathetic and parasympathetic activity, which may have implications for cardiovascular health [31]. Examining different sleep disorders and methods for measuring sleep fragmentation, Martynowicz et al. investigated the relationship between sleep fragmentation and cardiovascular risk [32]. They emphasized that when determining cardiovascular risk, it is crucial to consider both the length of sleep and its quality, especially its continuity. Parameters like the arousal burden (AB), sleep fragmentation index (SFI), and arousal index (ArI) may be useful to quantify and define sleep fragmentation. 

## 4. How Do Chemoreflex, Baroreflex, Inflammation, and Oxidative Stress Contribute to Sympathetic Overactivity in OSA?

Several pathways have been proposed to explain the link between OSA and increased sympathetic nervous system activity. One of the primary mechanisms involves the chemoreflex, a feedback system that regulates ventilation in response to changes in blood oxygen and carbon dioxide levels [33]. In OSA, the recurrent episodes of hypoxia and hypercapnia during apneic events can lead to chemoreflex hypersensitivity and a sustained increase in sympathetic outflow, even during wakefulness [34]. Additionally, the intermittent hypoxia associated with OSA has been shown to enhance carotid body sensitivity, further contributing to chemoreflex-mediated sympathetic activation [35]. Another important mechanism linking OSA to sympathetic overactivity involves the baroreflex, a homeostatic feedback system that regulates blood pressure by modulating heart rate and vascular tone [36]. In healthy individuals, the baroreflex helps to maintain stable blood pressure by increasing sympathetic activity when blood pressure drops and decreasing sympathetic activity when blood pressure rises [37]. However, in patients with OSA, the recurrent episodes of hypoxia and arousal can lead to baroreflex dysregulation, characterized by a blunted baroreflex sensitivity and a shift in the baroreflex set point to higher blood pressure levels [38,39]. This impairment in baroreflex function may contribute to the sustained increase in sympathetic activity and the development of hypertension in patients with OSA. Inflammation and oxidative stress have also been implicated in the pathways linking OSA to sympathetic overactivity and cardiovascular risk. The intermittent hypoxia and reoxygenation associated with OSA can lead to the generation of reactive oxygen species (ROS) and the activation of pro-inflammatory pathways, including nuclear factor-kappaB (NF-κB) and hypoxia-inducible factor-1 (HIF-1) [40,41]. These pathways can promote the release of inflammatory cytokines, such as tumor necrosis factor-alpha (TNF-α) and interleukin-6 (IL-6), which have been shown to contribute to endothelial dysfunction and atherosclerosis [42]. Moreover, inflammation and oxidative stress can also modulate sympathetic nervous system activity through their effects on central and peripheral neural pathways [43,44]. The interplay between inflammation, oxidative stress, and sympathetic activation in OSA may thus create a vicious cycle that exacerbates cardiovascular risk in these patients.

Recent studies have shown a growing interest in the impact of vitamin D on hypertension and sleep disturbances. Numerous physiological systems, such as blood pressure regulation and sleep-wake cycles, are known to be influenced by vitamin D. Kanclerska et al. investigated the relationship between AH and OSA by examining sleep architecture, vitamin D concentration, and electrolyte levels in patients with these coexisting conditions in 133 patients [45]. The authors did not find any statistically significant variations in vitamin D content between hypertensive and normotensive individuals with suspected OSA. This finding is at odds with the other, earlier studies that proposed links between low vitamin D levels and OSA or hypertension. But the connection between vitamin D, sleep, and hypertension is still complicated and can be impacted by a number of different things. Although its benefits on sleep quantity and problems were less obvious, vitamin D supplementation showed promise in enhancing the quality of sleep according to a comprehensive review and meta-analysis by Abboud et al. [46]. In a similar vein, Mirzaei-Azandaryani et al.’s meta-analysis showed that vitamin D administration significantly improved the quality of sleep [47]. Remarkably, a more recent systematic review and meta-analysis on OSA and vitamin D by Loh et al. found that individuals with OSA had a greater frequency of vitamin D deficiency and considerably lower serum 25-hydroxyvitamin D levels than those without OSA [48]. This correlation was especially strong in the instances of moderate to severe OSA and was unaffected by age or BMI. The difference in these results from those of Kanclerska et al. emphasizes the need for more investigation to completely clarify the function of vitamin D in the pathophysiology of OSA and hypertension, taking into account possible confounding variables and the intricate interactions between different biochemical markers.

## 5. What Is the Impact of Sympathetic Overactivity on Cardiovascular Health?

Sympathetic overactivity has been identified as a key contributor to the development and maintenance of hypertension in patients with obstructive sleep apnea [45]. The recurrent episodes of hypoxia and arousal in OSA can lead to the persistent activation of the sympathetic nervous system, resulting in increased peripheral vascular resistance and elevated blood pressure [49]. Moreover, the chronic intermittent hypoxia associated with OSA has been shown to impair endothelial function and promote vascular inflammation, further exacerbating the hypertensive state [50]. Studies have demonstrated that continuous positive airway pressure (CPAP) treatment, the gold standard for OSA management, can significantly reduce sympathetic activity and improve blood pressure control in patients with OSA [51,52]. Sympathetic overactivity has also been implicated in the pathogenesis of cardiac arrhythmias in patients with OSA [53]. The recurrent hypoxia and reoxygenation episodes in OSA can lead to oxidative stress and inflammation, which may alter the electrophysiological properties of the myocardium and increase the risk of arrhythmias [54]. Additionally, the increased sympathetic tone associated with OSA can promote the development of atrial and ventricular arrhythmias, particularly atrial fibrillation [55]. Studies have shown that patients with OSA have a higher prevalence of atrial fibrillation compared to the general population, and that CPAP treatment can reduce the recurrence of atrial fibrillation in these patients [56,57]. Chronic sympathetic overactivity in patients with OSA can also contribute to myocardial remodeling and dysfunction [58]. The increased workload imposed on the heart by the elevated sympathetic tone and the recurrent episodes of hypoxia can lead to left ventricular hypertrophy and diastolic dysfunction [59]. Over time, these structural and functional changes can progress to more severe forms of cardiac dysfunction, such as heart failure [60]. Additionally, the chronic intermittent hypoxia associated with OSA has been shown to promote myocardial fibrosis and apoptosis, further contributing to the deterioration of cardiac function [61]. CPAP treatment has been demonstrated to attenuate myocardial remodeling and improve cardiac function in patients with OSA [62,63]. Sympathetic overactivity in patients with OSA can also have significant cerebrovascular consequences [64]. The chronic intermittent hypoxia and the recurrent surges in blood pressure associated with OSA can lead to endothelial dysfunction and the development of atherosclerotic plaques in the cerebral vasculature [65]. These changes can increase the risk of stroke and other cerebrovascular events in patients with OSA [66]. Moreover, the increased sympathetic tone and the altered cerebral blood flow regulation in OSA can contribute to cognitive impairment and the development of vascular dementia [67,68]. CPAP treatment has been shown to improve cerebral blood flow and cognitive function in patients with OSA, highlighting the importance of addressing sympathetic overactivity in this population [69,70,71].

## 6. What Are the Relevant Diagnostic Approaches to Assess Sympathetic Activity in the Case of OSA?

Microneurography, a technique that directly records muscle sympathetic nerve activity (MSNA) using intraneural electrodes, is considered the gold standard for assessing sympathetic tone in humans [72]. MSNA provides a direct measure of efferent sympathetic nerve traffic to the skeletal muscle vasculature, which is a key determinant of peripheral vascular resistance and blood pressure [73]. Presently, MSNA measurements represent the gold standard for the assessment of sympathetic activity in patients with OSA. Studies using microneurography have consistently demonstrated elevated MSNA in patients with OSA compared to healthy controls, both during sleep and wakefulness [74,75]. Moreover, the severity of OSA, as measured by the apnea/hypopnea index (AHI), has been shown to correlate with the degree of MSNA elevation [76]. CPAP treatment has been found to reduce MSNA in patients with OSA, further supporting the role of OSA in sympathetic overactivity [77,78]. Measuring the plasma or urinary levels of norepinephrine and other catecholamines is a common approach to assessing sympathetic activity in patients with obstructive sleep apnea [79]. Norepinephrine, a key neurotransmitter released by sympathetic nerve endings, is often used as a surrogate marker for sympathetic tone [80]. Studies have shown that patients with OSA have elevated levels of norepinephrine compared to healthy controls, and that the severity of OSA correlates with the degree of norepinephrine elevation [81,82]. Additionally, the successful treatment of OSA with CPAP has been demonstrated to reduce norepinephrine levels, suggesting a causal relationship between OSA and sympathetic overactivity [83,84]. Heart rate variability (HRV) analysis is another widely used method to assess autonomic nervous system function, including sympathetic activity, in patients with OSA [85]. HRV refers to the physiological variation in the time interval between consecutive heartbeats, which is influenced by the balance between sympathetic and parasympathetic tone [86]. In patients with OSA, recurrent episodes of hypoxia and arousal can lead to a shift in autonomic balance towards sympathetic predominance, resulting in reduced HRV [87]. The spectral analysis of HRV can provide insights into the relative contributions of sympathetic and parasympathetic activity, with the low-frequency component (LF) reflecting a mix of both influences and the high-frequency component (HF) primarily representing parasympathetic modulation [88]. Studies have shown that patients with OSA have a higher LF/HF ratio compared to healthy controls, indicating increased sympathetic activity [89,90]. It is noteworthy that the assessment of sympathetic activity in individuals with OSA is predominantly an experimental procedure and is not customarily carried out in medical settings. Sympathetic function is mainly measured in research settings in order to analyze the impact of different therapies on sympathetic activity and to gain a better understanding of the mechanisms that link OSA to cardiovascular risk. Instead of using direct measurements of sympathetic activity, the diagnosis and treatment of OSA in clinical settings usually depend on sleep studies, symptom assessment, and the assessment of related comorbidities. Assessing baroreflex sensitivity (BRS) is another approach to evaluating sympathetic function in patients with OSA [91,92]. In healthy individuals, an increase in blood pressure leads to a reflex-mediated decrease in heart rate and sympathetic outflow, while a decrease in blood pressure has the opposite effect [93]. However, in patients with OSA, recurrent episodes of hypoxia and arousal can impair baroreflex function, leading to a blunted BRS and a shift towards sympathetic predominance [94,95]. BRS can be assessed using various methods, including the sequence technique, which involves the analysis of spontaneous fluctuations in blood pressure and heart rate, and the spectral analysis of HRV and blood pressure variability [96]. Studies have shown that patients with OSA have reduced BRS compared to healthy controls, and that CPAP treatment can improve BRS, highlighting the impact of OSA on sympathetic regulation [97,98].

## 7. What Therapeutic Interventions Can Influence the Sympathetic Nervous System?

Continuous positive airway pressure (CPAP) therapy is the gold standard treatment for OSA and has been shown to effectively reduce sympathetic activity in these patients [99]. CPAP works by delivering a constant stream of air pressure through a mask worn during sleep, preventing the collapse of the upper airway and maintaining airflow [100]. Studies have demonstrated that CPAP treatment can significantly decrease MSNA and plasma norepinephrine levels in patients with OSA, indicating a reduction in sympathetic tone [101,102]. Moreover, CPAP has been shown to improve HRV and BRS, further supporting its role in modulating autonomic function [103,104]. The reduction in sympathetic activity achieved with CPAP therapy may contribute to the improvement of various cardiovascular outcomes in patients with OSA, such as blood pressure control and the reduction in arrhythmia risk [105,106]. Oral appliance therapy is an alternative treatment option for patients with OSA who are unable to tolerate CPAP or have mild to moderate disease severity [107]. Oral appliances, such as mandibular advancement devices (MADs), work by repositioning the lower jaw and tongue forward, thereby increasing the size of the upper airway and reducing the likelihood of collapse during sleep [108]. While the effects of oral appliance therapy on sympathetic activity have not been as extensively studied as those of CPAP, some evidence suggests that MADs can reduce sympathetic tone in patients with OSA [109,110]. A study by Itzhaki et al. found that MAD treatment significantly decreased urinary norepinephrine levels and improved HRV parameters, indicating a reduction in sympathetic activity [111]. However, more research is needed to fully elucidate the impact of oral appliance therapy on the sympathetic nervous system in patients with OSA. It is crucial to stress that although OSA treatments have been demonstrated in experimental procedures to reduce sympathetic activity, the main goal of adopting these treatments in clinical practice is to lessen the symptoms and negative health effects of OSA. The main goals of treatments including continuous positive airway pressure (CPAP), dental appliances, and surgical procedures are to lessen daytime drowsiness, enhance the quality of sleep, and lessen the hazards to the heart and metabolism that come with untreated OSA. Additionally, treating OSA is crucial for managing comorbidities such as hypertension, arrhythmias, and insulin resistance. While a positive consequence, the decrease in sympathetic activity is not the primary clinical reason to start OSA treatment.

## 8. What Role Do Pharmacological Treatments Play in Managing OSA and Sympathetic Nervous System?

Pharmacological agents that target the sympathetic nervous system have been explored as potential adjunctive therapies for patients with OSA [112]. One such agent is clonidine, an alpha-2 adrenergic agonist that acts centrally to reduce sympathetic outflow [113]. A study by Heitmann et al. found that a single dose of oral clonidine significantly decreased MSNA and blood pressure in patients with OSA, suggesting its potential utility in reducing sympathetic overactivity [114]. Another pharmacological approach involves the use of beta-blockers, which block the effects of norepinephrine on beta-adrenergic receptors, thereby attenuating sympathetic influence on the heart and blood vessels [115]. While beta-blockers have been shown to improve cardiovascular outcomes in patients with OSA, their direct effects on sympathetic activity remain to be fully elucidated [116,117]. It is important to note that pharmacological interventions for OSA should be used cautiously and under close medical supervision, as they may have potential side effects and interactions with other medications. Addressing sympathetic activity in OSA directly with pharmaceutical therapy is not the norm. Rather, these drugs are usually administered to treat comorbid illnesses including hypertension and heart disease, which frequently co-occur with OSA. In order to manage hypertension and enhance cardiovascular outcomes in patients with OSA, for instance, ACE inhibitors may be administered in addition to beta-blockers to lower blood pressure and lessen the strain on the heart. Complementary conditions and the patient’s overall cardiovascular and metabolic risk profile dictate when and how antihypertensive, antiarrhythmic, and glucose-lowering drugs are used. Lifestyle modifications, particularly weight loss, have been shown to effectively reduce sympathetic activity in patients with OSA [118]. Obesity is a major risk factor for OSA, and the excess adipose tissue can contribute to sympathetic overactivity through various mechanisms, such as increased leptin levels and insulin resistance [119]. Studies have demonstrated that weight loss achieved through dietary modifications and exercise can significantly decrease MSNA and plasma norepinephrine levels in obese patients with OSA [120,121]. Moreover, weight loss has been shown to improve HRV and BRS, indicating a beneficial effect on autonomic function [121,122]. In addition to its direct impact on sympathetic activity, weight loss can also improve OSA severity by reducing the mechanical load on the upper airway, further contributing to the reduction in sympathetic tone [123,124]. Therefore, lifestyle modifications aimed at weight loss should be strongly encouraged as a key component of the comprehensive management of OSA and its associated sympathetic overactivity. In recent years, there has been research in the relationship between vitamin D and both sleep quality and obstructive sleep apnea (OSA). Numerous research have looked into the possible connection between OSA severity, vitamin D levels, and sleep characteristics (Table 1). The results, however, have been conflicting, which has sparked ongoing debates among scientists. In comparison to non-OSA controls, vitamin D deficiency was demonstrated common in OSA patients, particularly in those with moderate-to-severe OSA, and that OSA patients’ serum vitamin D levels were significantly lower [49]. Although the exact causal association between the severity of OSA and vitamin D level is yet unknown, our finding suggests a possible correlation.

## 9. What Are the Future Directions and Research Priorities?

As our understanding of the complex interplay between OSA, sympathetic activation, and cardiovascular risk continues to evolve, several key areas have emerged as future directions and research priorities. The development and refinement of emerging technologies for sympathetic assessment, such as non-invasive imaging techniques and wearable devices, hold promise for improving the accuracy and accessibility of sympathetic monitoring in patients with OSA [125]. These advancements may enable the early detection of sympathetic dysregulation and facilitate timely interventions to prevent cardiovascular complications [126]. Additionally, exploring novel therapeutic targets for sympathetic modulation is a crucial avenue for future research. This includes investigating the potential of neuromodulation techniques, such as renal denervation or baroreceptor stimulation, as adjunctive therapies for managing sympathetic hyperactivity in OSA [127]. Furthermore, the identification of specific molecular pathways and genetic factors that contribute to sympathetic dysregulation in OSA may open new doors for targeted pharmacological interventions [128]. Lastly, long-term studies on cardiovascular outcomes in patients with OSA are essential to fully understand the impact of sympathetic activation on cardiovascular morbidity and mortality [129]. These studies should focus on evaluating the effectiveness of various treatment strategies, including CPAP therapy, pharmacological interventions, and lifestyle modifications, in mitigating cardiovascular risk and improving long-term outcomes [129]. By addressing these research priorities and leveraging emerging technologies and therapeutic approaches, we can work towards developing more effective and personalized strategies for managing sympathetic activation and reducing cardiovascular risk in patients with OSA [130]. The social and environmental milieu in which people reside has a significant impact on the health-related behaviors and the consequences they experience. Promoting health and well-being at the individual and population levels requires a multifaceted approach that takes into account the intricate interactions between social inequalities, economic factors, politics, and the physical environment, as Leischik et al. points out in their article “Plasticity of Health” [131]. Urban planning, social capital, education, socioeconomic status, and ecosystem health are just a few examples of the variables that can have a big impact on people’s capacity to practice healthy habits and get the treatment and resources they need. In order to create supportive environments where everyone may reach their full potential for health, it is imperative that these upstream determinants of health be addressed through intersectoral collaboration and policies that promote health. Developing practical, long-lasting plans to lower health disparities and enhance well-being throughout life requires incorporating this larger view of how health is malleable and influenced by social and environmental factors.

## 10. Conclusions

This review has highlighted the critical role of OSA-induced sympathetic activation in the pathogenesis of cardiovascular disease. The key findings emphasize the complex interplay between intermittent hypoxia, oxidative stress, inflammation, and autonomic dysregulation in promoting cardiovascular risk in patients with OSA. The clinical implications of these findings underscore the importance of the early detection and appropriate management of OSA to mitigate its deleterious effects on cardiovascular health. Clinical recommendations include the use of sympathetic markers for risk stratification, personalized treatment approaches based on sympathetic profiles, and the combination of OSA and cardiovascular therapies for optimal outcomes. Future research directions should focus on the development of novel technologies for sympathetic assessment, exploration of targeted therapeutic interventions for sympathetic modulation, and long-term studies to evaluate the impact of OSA treatment on cardiovascular morbidity and mortality. By addressing these research priorities and translating the findings into clinical practice, we can work towards improving cardiovascular outcomes and quality of life for individuals with OSA.

## Figures and Tables

**Table 1:** Characteristics of included studies. Abbreviations: OSA, obstructive sleep apnea; N.A., Not Applicable; arousal burden, AB; arousal index, ArI; sleep fragmentation index, SFI.

Study	Year	Country	Sample Size	Study Design	Main Results	Study Limitations
Martynowicz et al. [32]	2024	Poland	-	Examined relationship between sleep fragmentation and cardiovascular risk	Sleep fragmentation parameters like AB, SFI, and ArI may be useful to quantify sleep fragmentation and determine cardiovascular risk	Study design not adequate
Kanclerska et al. [45]	2024	Poland	133 patients	Investigated relationship between AH and OSA by examining sleep architecture, vitamin D, and electrolyte levels	No significant differences in vitamin D levels found between hypertensive and normotensive OSA patients, contrasting previous studies	the study did not comprehensively examine the potential influence of calcium, magnesium, vitamin D and uric acid concentrations on the sleep architecture of patients with comorbid arterial hypertension and obstructive sleep apnea
Abboud et al. [47]	2022	United Arab Emirates	19 studies	Systematic review and meta-analysis	Vitamin D supplementation showed promise in improving sleep quality but had less clear effects on sleep quantity and disorders	Limitations of included studies not discussed in the review
Mirzaei-Azandaryani et al. [48]	2022	Iran	18 studies	Meta-analysis	Vitamin D administration significantly improved sleep quality	Limitations of included studies not discussed in the review
Loh et al. [49]	2023	Malaysia	18 studies	Systematic review and meta-analysis	OSA patients had higher prevalence of vitamin D deficiency and significantly lower serum vitamin D levels vs. non-OSA controls, especially in moderate-severe OSA	Limitations of included studies not discussed in the review

## Data Availability

All the data reported are present on PubMed, Embase, and Web of Science web database.
